# The complete mitochondrial genome of the spotted knifejaw *Oplegnathus punctatus*

**DOI:** 10.1080/23802359.2023.2292737

**Published:** 2023-12-21

**Authors:** Bo Gao, Shuran Du, Fei Zhu, Dafeng Xu, Qian Meng, Chaofeng Jia, Ruijian Sun

**Affiliations:** a Marine Fisheries Research Institute of Jiangsu Province, Nantong, China; b Jiangsu Key Laboratory for Genetics and Breeding of Marine Fishes, Nantong, China

**Keywords:** Oplegnathus punctatus, mitochondrial genome, spotted knifejaw, Perciformes, marine fish

## Abstract

In this study, the mitochondrial genome was sequenced in a new commercial species, spotted knifejaw (*O. punctatus*), using next-generation sequencing and PCR-based methods. The overall length of the female *O. punctatus* mitochondrial genome was 16,508 bp. It contained 13 PCGs, 2 r-RNA genes, 22 t-RNA genes, and a displacement loop locus (a control region). The total nucleotide composition was 28.75% A, 25.69% T, 29.70% C, and 15.86% G, with a total A + T content of 54.44%. The results demonstrated that the mitochondrial genome of *O. punctatus* has a high sequence identity with that of another species of Perciformes. This finding provides a deeper understanding of mitogenomic diversity and evolution in marine fish.

## 1. Introduction

**Figure 1.**
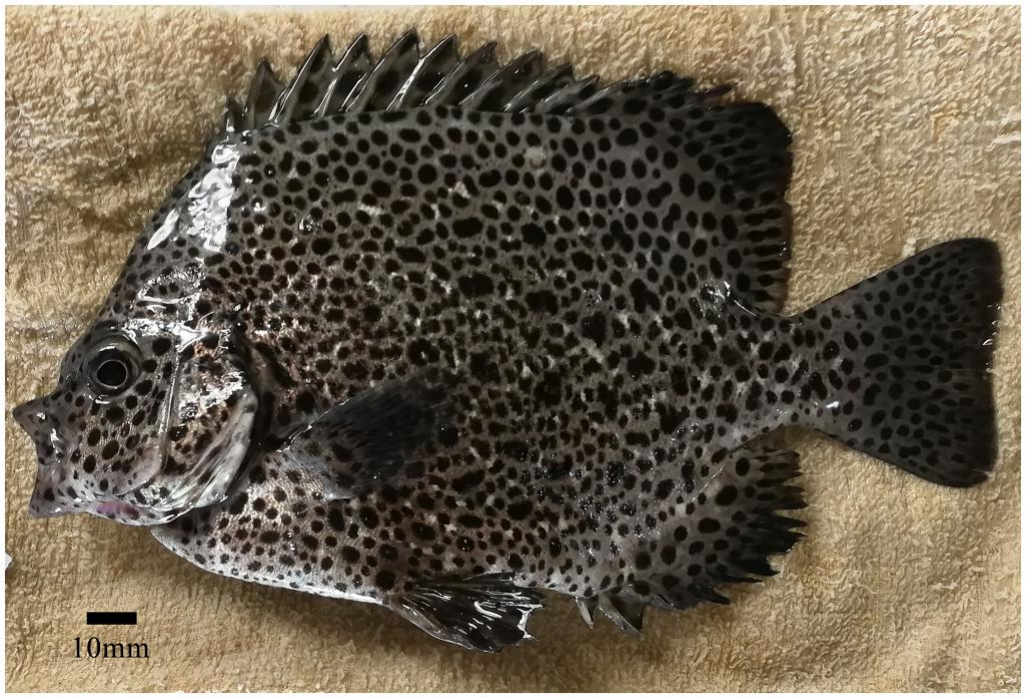
Specimen of Oplegnathus punctatus was obtained from Yangtze River estuary, China (31.67°N, 121.86°E). photographs of Oplegnathus punctatus at 4 month by Fei Zhu and Qian Meng on Aug 10, 2019.

In the northwest and center of the Pacific Ocean, spotted knifejaw (*O. punctatus*) (Temminck and Schlegel, 1844), which is mostly found in subtropical and mild temperate seas, is a promising new commercial species in China, Japan, and Korea because of its high nutritional value and limited supply, and it might become a widely cultivated fish in the aquaculture industry in the future (Froese and Pauly 2021; GBIF 2021; Jia et al. 2021). Its market demand has been constantly increasing in recent years, but resources are limited (Wang et al. 2021). The total length of *O. punctatus* is estimated at approximately 50 cm, the single dorsal fin shows a pattern of tXI-XII,11-24, and the anal fin shows a pattern of III,11-17. *O. punctatus* mainly swim in rocky and coral reef areas at depths ranging from 2 to 5 meters (Yusuf et al. 2021). Diagnostic features of *O. punctatus* are multiple irregular dark spots on the head, body, dorsal fins, anal fins, and caudal fins. Its head and body are grayish brown with whitish jaws, and small ctenoid scales cover the body (Kimura 2018). In adults, the jaw teeth are fused into a parrot-like beak (Froese and Pauly 2021). The reference image was taken by Fei Zhu and Qian Meng on Aug 10, 2019 (Figure 1).

Mitochondrial DNA consists of 16 to 18 kbp and is circular in vertebrates. It contains 13 PCGs, 2 ribosomal RNA genes, 22 transfer RNA genes, and one displacement loop locus (a control region) in humans (Anderson et al. 1981). Mitochondrial DNA is widely used as a population genetic marker for gene flow and hybridization inference because of its compactness and evolutionary rate, which is faster than that of nuclear DNA (Moore 1995). Since 1992, more than 300 mitochondrial DNA sequences of species within Teleostei have been sequenced (Oh et al. 2007). However, no complete mitogenome of *O. punctatus* is currently available, limiting our research on its evolution. Here, we assembled and annotated the complete mitochondrial genome of spotted knifejaw and compared it with those of other Perciformes species.

## 2. Materials and methods

### 2.1. Sample collection and preservation

Spotted knifejaw samples were obtained from the Yangtze River Estuary, China (31.67°N, 121.86°E), with permission from the Marine Fisheries Research Institute of Jiangsu Province. Voucher photographs are provided in Figure 1. Eugenol (0.2 ml/l) was used for the anesthesia of the samples. An approximately 50 mg fin clip was taken from each fish with sterile scissors and stored in ethanol. Total genomic DNA was extracted from these caudal fins by a Tissue DNA Kit (Aidlab Biotechnologies Co., Ltd., China) following the manufacturer’s protocol. These tissues were deposited in the Marine Fisheries Research Institute of Jiangsu Province (http://kxjst.jiangsu.gov.cn/col/col83421/index.html; Fei Zhu; ebancool@126.com； voucher number: YZS-ZF-191025opy-11).

### 2.2. DNA extraction, sequencing, and assembly

The extraction was performed using a DNA Rapid Extraction Kit (Beijing Aidlab Biotechnologies Co., Ltd.) according to the kit manual. The integrity of the DNA samples was detected by agarose gel electrophoresis and a NanoDrop 2000 spectrophotometer (Thermo Fisher Scientific, USA). The DNA was diluted to a concentration of approximately 100 ng/μl and then stored at −20 °C. Primers (Table 1) were designed according to the mt genomic sequences of closely related species, and long PCR (LA-PCR) amplification was performed using LA Taq polymerase. The PCR products were sequenced directly.

**Table 1.** Characteristics of the mitochondrial genome of O. punctatus.

Gene Name	Start	Stop	Length	Space (+) Overlap (−)	Codons	
					Initial/Terminal	Strand
trnF(ttc)	1	68	68	0		+
rrnS	69	1019	951	1		+
trnV(gta)	1021	1092	72	1		+
rrnL	1094	2790	1697	0		+
trnL2(tta)	2791	2864	74	15		+
nad1	2880	3830	951	13	ATG TAA	+
trnI(atc)	3844	3913	70	−1		+
trnQ(caa)	3913	3983	71	−1		–
trnM(atg)	3983	4051	69	0		+
nad2	4052	5089	1038	8	ATG TAA	+
trnW(tga)	5098	5168	71	1		+
trnA(gca)	5170	5238	69	1		–
trnN(aac)	5240	5312	73	39		–
trnC(tgc)	5352	5416	65	0		–
trnY(tac)	5417	5487	71	7		–
cox1	5495	7027	1533	12	GTG TAA	+
trnS2(tca)	7040	7110	71	3		–
trnD(gac)	7114	7186	73	6		+
cox2	7193	7876	684	7	ATG T	+
trnK(aaa)	7884	7957	74	4		+
atp8	7962	8126	165	−7	ATG TAA	+
atp6	8120	8800	681	2	ATG TAA	+
cox3	8803	9585	783	2	ATG TAA	+
trnG(gga)	9588	9659	72	0		+
nad3	9660	10,007	348	1	ATG TAG	+
trnR(cga)	10,009	10,077	69	0		+
nad4l	10,078	10,371	294	−4	ATG TAA	+
nad4	10,368	11,741	1374	7	ATG T	+
trnH(cac)	11,749	11,817	69	0		+
trnS1(agc)	11,818	11,885	68	4		+
trnL1(cta)	11,890	11,962	73	18		+
nad5	11,981	13,789	1809	11	ATG TAA	+
nad6	13,801	14,319	519	0	CTA CAT	–
trnE(gaa)	14,320	14,388	69	4		–
cob	14,393	15,526	1134	7	ATG T	+
trnT(aca)	15,534	15,605	72	−1		+
trnP(cca)	15,605	15,675	71	833		–

### 2.3. Annotation and analysis

The samples were annotated largely as described previously (Zou et al. 2017; Zhang et al. 2018). Briefly, sequenced fragments were quality-checked by visually inspecting the electropherograms and queried against GenBank using BLAST to confirm that the amplicon was the target sequence. The complete mt genome sequence was assembled from the sequenced fragments using DNAstar software (Burland 2000). We ensured that the overlaps between sequences were identical, the genome was circular, and that no numts (Hazkani-Covo et al. 2010) were incorporated. ORFs for PCGs were located using DNAstar and manually fine-tuned *via* a comparison with available sciaenid orthologs using BLAST and BLASTx. tRNAscan (Schattner et al. 2005) and ARWEN (Laslett and Canbäck 2008) were used to identify tRNAs. The mitochondrial DNA sequence of *O. punctatus* was annotated and verified by the MITOS WebServer (http://mitos.bioinf.uni-leipzig.de/index.py) (Bernt et al. 2013). Organelle genome maps were drawn by OGDRAW (https://chlorobox.mpimp-golm.mpg.de/OGDraw.html) (Greiner et al. 2019).

## 3. Results and discussion

### 3.1. Genomic characterization

**Figure 2.**
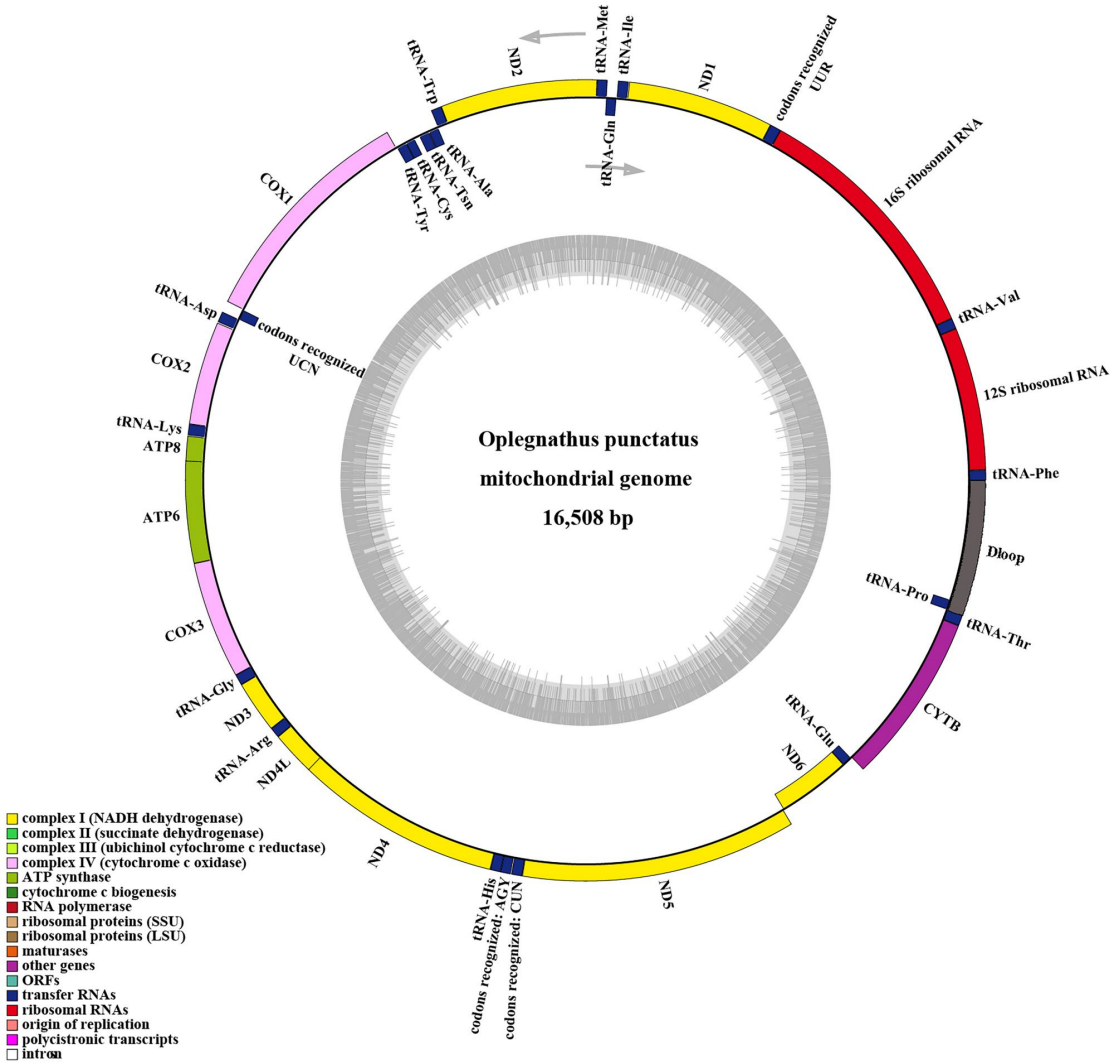
The complete mitochondrial genome map of O. punctatus (GenBank accession no. MN927588). the outermost circle genes are transcribed clockwise and the rest counter clockwise.

The total length of the mitochondrial genome for female *O. punctatus* was 16,508 bp (GenBank, accession no. MN927588). It contained 13 PCGs, 2 rRNAs, 22 tRNAs, and one control region. The overall nucleotide composition was 28.75% A, 29.70% C, 25.69% T, and 15.86% G, with a total A + T content of 54.44%. Nad6 and eight tRNA genes were encoded on the light chain, and other coding genes were encoded on the heavy chain (H-strand) (Table 1 and Figure 2).

### 3.2. Phylogenetic analysis

**Figure 3.**
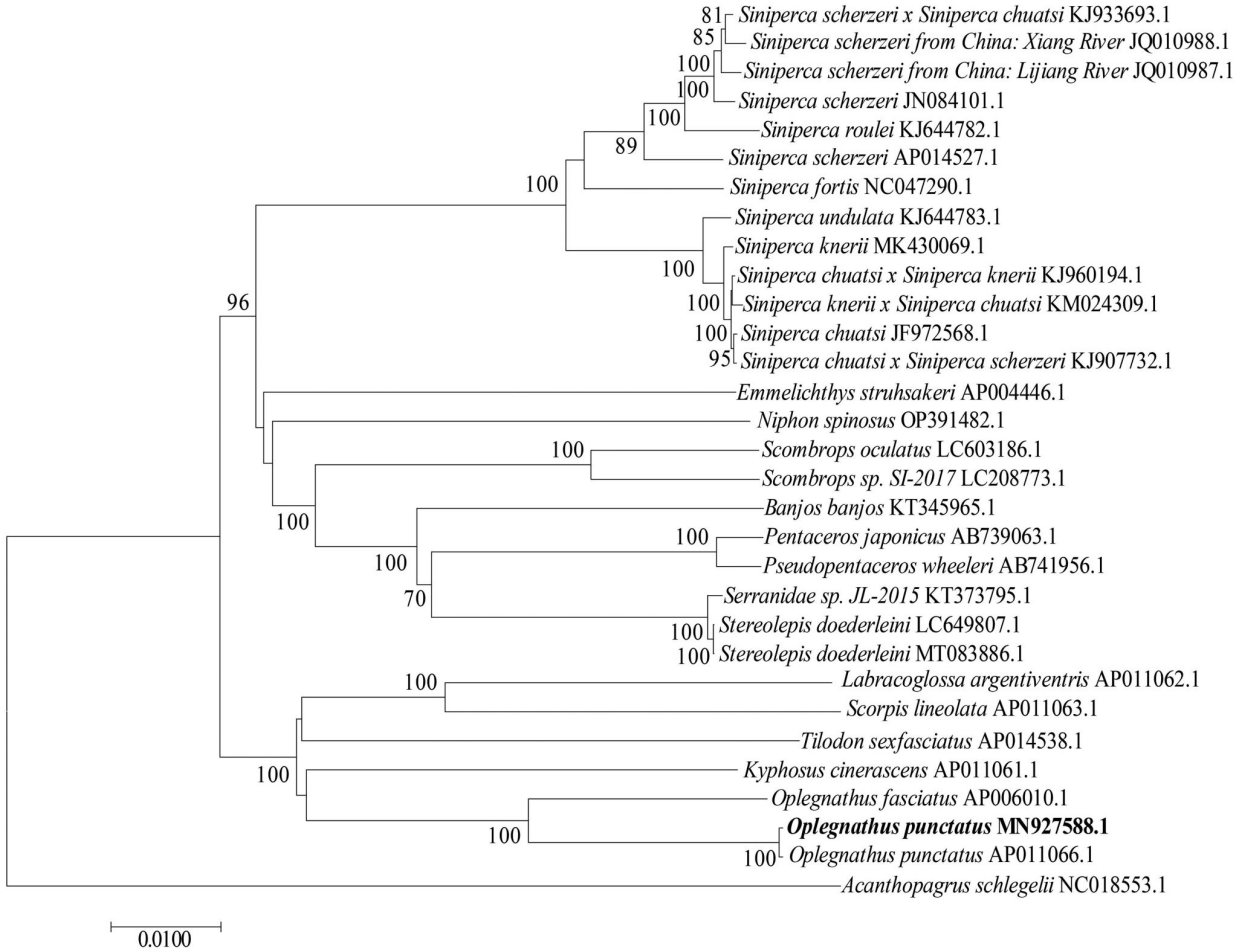
The phylogenetic analysis of mitochondrial genome of O. punctatus with other species. Phylogenetic tree was established by MEGA7 with neighbor-joining method. GenBank accession numbers are given adjacent to the species name. Scale bar indicates groupings of species into families. The taxa in bold is the mitogenome from this study. The numbers under the node indicate the bootstrap value as percentages obtained for 1000 replicates.

A homology search of the mitochondrial genomic datasets of *O. punctatus* was performed using the National Center for Biotechnology Information database (http://www.ncbi.nlm.nih.gov/). *O. punctatus* (MN927588.1), *O. punctatus* (AP011066.1) and *Oplegnathus fasciatus* (AP006010.1) belong to Sparidae, clustered on a large branch with *Kyphosus cinerascens* (AP011061.1)*, Tilodon sexfasciatus* (AP014538.1), *Scorpis lineolate* (AP011063.1) and *Labracoglossa argentiventris* (AP011062.1). *Stereolepis doederleini* (7.1&LC649807.1), *Serranidae sp.* JL-2015 (KT373795.1), *Pseudopentaceros wheeleri* (AB741956.1)*, Pentaceros japonicus* (AB739063.1), *Banjos banjos (KT345965.1)*, *Scombrops sp.* SI-2017 (LC208773.1), *Scombrops oculatus* (LC603186.1), *Niphon spinosus* (OP391482.1), *Emmelichthys struhsakeri* (AP004446.1)*, Siniperca chuatsi × Siniperca scherzeri* (KJ907732.1)*, Siniperca chuatsi* (JF972568.1)*, Siniperca knerii × Siniperca chuatsi* (KM024309.1), *Siniperca chuatsi × Siniperca knerii* (KJ960194.1)*, Siniperca knerii* (MK430069.1)*, Siniperca roulei* (KJ644782.1)*, Siniperca scherzeri* (JN084101.1)*, Siniperca scherzeri from China*: *Lijiang River* (JQ010987.1)*, Siniperca scherzeri from China*: *Xiang River* (JQ010988.1) and *Siniperca scherzeri × Siniperca chuatsi* (KJ933693.1), belonging to Perciformes, clustered on a large branch with the previous branch. *Acanthopagrus schlegelii* (NC018553.1) was included an outgroup (Figure 3).

## 4. Conclusion

In conclusion, this study is the first to report the complete mitochondrial genome of spotted knifejaw (*O. punctatus*) (GenBank, accession no. MN927588). The molecule was 16,508 bp long and showed a circular structure typical of vertebrate mitogenomes. Phylogenetic analysis revealed that the mitochondrial genome of *O. punctatus* as a sister to *O. punctatus* (AP011066.1) and *Oplegnathus fasciatus* (AP006010.1), belonged to Sparidae. The complete mitochondrial genome sequence of *O. punctatus* provides a deeper understanding of mitogenomic diversity and evolution in marine fish. In the future, we could develop novel genetic markers to facilitate population genetics studies and species identification.

## Data Availability

The genome sequence data that support the findings of this study are openly available in GenBank of NCBI at https://www.ncbi.nlm.nih.gov/ under the accession no. MN927588.1.
